# Differentiation and CRISPR-Cas9-mediated genetic engineering of human intestinal organoids

**DOI:** 10.1016/j.xpro.2022.101639

**Published:** 2022-08-18

**Authors:** Adriana Martinez-Silgado, Fjodor A. Yousef Yengej, Jens Puschhof, Veerle Geurts, Charelle Boot, Maarten H. Geurts, Maarten B. Rookmaaker, Marianne C. Verhaar, Joep Beumer, Hans Clevers

**Affiliations:** 1 Hubrecht Institute, Royal Netherlands Academy of Arts and Sciences (KNAW) and UMC Utrecht, 3584 CT Utrecht, the Netherlands; 2 Department of Nephrology and Hypertension, University Medical Center Utrecht, 3584 CX Utrecht, the Netherlands; 3 Microbiome and Cancer Division, German Cancer Research Center (DKFZ), Im Neuenheimer Feld 280, 69120 Heidelberg, Germany; 4 Head of Pharma, Research and Early Development (pRED) and Member of the Enlarged Executive Committee of F. Hoffmann La Roche Ltd., Basel, Switzerland

**Keywords:** CRISPR, Cell differentiation, Organoids

## Abstract

Intestinal organoids are three-dimensional cultures that resemble key aspects of the epithelium of origin. Here, we describe how to differentiate human small intestinal organoids by combining growth media variations and genetic engineering. We detail the differentiation of human intestinal organoids in the presence and absence of BMP agonists to recapitulate a broader scope of functional cell states found *in vivo*. Using transient overexpression of the transcription factor Neurogenin-3, we describe the enhancement of differentiation toward rare enteroendocrine cells.

For complete details on the use and execution of this protocol, please refer to Beumer et al. (2022).

## Highlights

• Differentiation of human small intestinal organoids by growth media variations
• BMP-mediated functional specialization of intestinal lineages
• CRISPR/Cas9-mediated loss of function experiments in human small intestinal organoids
• Generation of enteroendocrine-cell enriched organoids via NEUROG3 overexpression

## Abstract (editor highlights)

Publisher’s note: Undertaking any experimental protocol requires adherence to local institutional guidelines for laboratory safety and ethics.

## Graphical abstract

**Figure:**
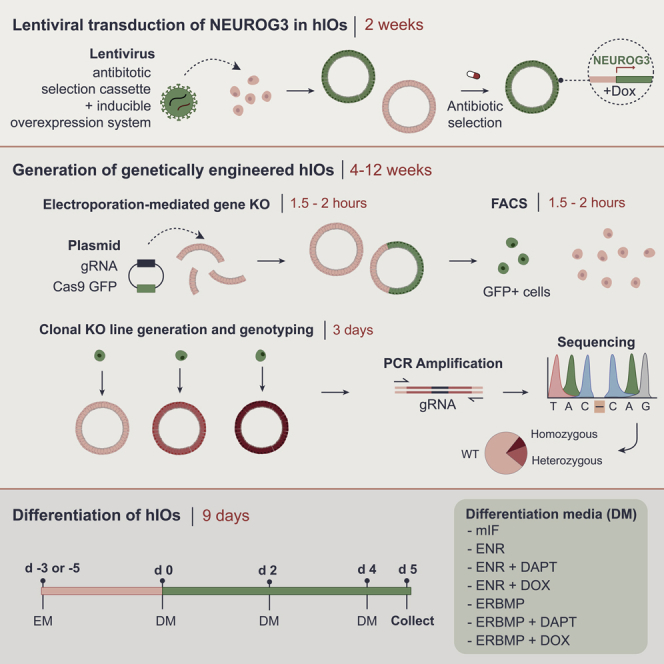

## Before you begin

The intestinal epithelium is organized in units of crypts and villi. Adult stem cells (ASCs) in the intestinal crypts continuously proliferate to renew the complete epithelium. Daughter cells travel from the crypt bottom to the villus tip - where these are shed - differentiating towards effector cells during this journey. These include nutrient absorbing enterocytes and secretory lineage cell types such as niche factor-secreting Paneth cells, mucus-producing goblet cells, hormone-secreting enteroendocrine cells (EECs) and tuft cells that regulate mucosal immunity. The changing environment along the crypt-villus axis guides both differentiation and functional subspecialization of differentiated cells. (Beumer et al., 2018, 2022; Moor et al., 2018; Beumer and Clevers, 2021).

Human intestinal organoids (hIOs) are three-dimensional multicellular epithelial cultures that mimic gut homeostatic renewal and function *in vitro* (Sato et al., 2009, 2011). In the past decade, organoids have proven valuable for studies of physiology and disease, drug screenings, personalized medicine and regenerative medicine (Yui et al., 2012; Clevers, 2016; Rossi et al., 2018; Berkers et al., 2019; Beumer et al., 2020). Differentiation conditions that mimic the *in vivo* niche, or overexpression of pivotal transcription factors, allow control over differentiation of intestinal organoids towards specific cell types and their functions (Sato et al., 2011; Yin et al., 2014; Basak et al., 2017; Beumer et al., 2018, 2020, 2022). This permits detailed investigation of specific cell types.

Here, we provide protocols for expansion of ASC-derived hIOs and differentiation towards enterocytes, goblet cells and EECs. The major intestinal lineages can be generated by simple manipulation of growth factors (Sato et al., 2011; Yin et al., 2014; Basak et al., 2017; Beumer et al., 2018, 2020, 2022; Fujii et al., 2018). Rare EECs can be enriched using a pulsed overexpression of the transcription factor Neurogenin-3 (NEUROG3), which provides a unique tool to study rare EECs *in vitro* (Beumer et al., 2020). We also describe CRISPR-Cas9-mediated genetic manipulation of hIOs, which enables evaluation of the role of specific proteins within these cell types in health and disease (Geurts et al., 2020; Hendriks et al., 2020; Beumer et al., 2021, 2022). These protocols were optimized using human ileal organoids, but can also be applied to other small intestinal organoid lines. Protocols for the generation of CRISPR-Cas9-induced knock-out lines in colonic organoids (using lentiviral transduction) have been reported as well (Gu et al., 2022). Together, these protocols present a toolbox that enables in-depth evaluation of the different cell types and functions of the human intestine *in vitro*.

### Institutional permissions

All procedures outlined in this protocol were carried out according with all relevant ethical regulations regarding research involving human participants, were approved by the UMC Utrecht (Utrecht, the Netherlands) ethical committee and were in accordance with the Declaration of Helsinki and according to Dutch law. Before any work with organoids derived from primary material is started, it is essential that institute and ethical approval is ensured and that subsequent experiments are following all relevant regulatory standards.

### gRNA design and cloning

**Timing: 3 days**

There are multiple online tools available that can aid in the design of gRNAs. These include Benchling (Benchling, 2022), CHOPCHOP (Labun et al., 2019), and ICE Synthego (Synthego Performance Analysis, 2019). We recommend selecting gRNAs that target an early exon to ensure complete disruption of the protein. Functional domains of a protein, such as transmembrane or enzymatic regions, could also be specifically targeted. When targeting early exons, it is important to exclude the use of alternative translational initiation sites. The resulting frameshift leads to a premature stop codon that causes the transcript degradation by non-sense mediated decay. It is desirable to design two gRNAs for each target in case one fails.

The protocol by Ran et al. provides instructions on how to clone the guide target sequence into the pSpCas9(BB)-2A-GFP PX458 backbone (Addgene 48138) (Ran et al., 2013).

### Genotyping primers design

**Timing: 15 min**

After transfection, it is necessary to amplify and sequence the region of interest to assess the efficacy of the CRISPR-mediated gene editing and identify clones carrying the desired mutation. For this, we use both PCR amplification primers and a separate genotyping primer. NCBI provides a tool for finding specific primers (PrimerBLAST) (Ye et al., 2012). Both the forward and reverse amplification primers should be at least 100 base pairs (bps) away from the gRNA, and we generally genotype using a PCR product of 450–600 bps. Designing a different primer for sequencing improves sequencing quality, due to reduced amplification of unspecific sequences. The sequencing primer should be at least 50 bps distant from the gRNA to avoid disturbed annealing to potentially deleted nucleotides, and should bind a region within the amplified product.

### Growth factor stocks preparation

**Timing: 30 min**

**Table:** 

Reagent	Final stock concentration	Amount	Volume
Nicotinamide	1 M	12.2 g	100 mL PBS
N-Acetyl-L-cysteine	500 mM	4 g	49 mL MilliQ
SB202190 (p38 inhibitor)	30 mM	25 mg	2.26 mL DMSO
EGF	500 μg/mL	1 mg	2 mL 0.1% BSA in PBS
A83-01 (ALK4/5/7 inhibitor)	5 mM	50 mg	23.7 mL DMSO
Prostaglandin E2	10 mM	100 mg	28.37 mL DMSO
Y-27632 dihydrochloride (Rho-kinase inhibitor)	10 mM	100 mg	31.2 mL MilliQ
BMP-2	50 μg/mL	100 μg	2 mL 0.1% BSA in PBS
BMP-4	50 μg/mL	100 μg	2 mL 0.1% BSA in PBS
DAPT	10 mM	25 mg	5.8 mL DMSO
IGF-1	100 μg/mL	100 μg	1 mL 0.1% BSA in PBS
FGF-2	50 μg/mL	50 μg	1 mL 0.1% BSA in PBS
Doxycycline	500 μg/mL	50 mg	100 mL 70% Ethanol

Store at −20°C for up to 2 months. Prepare aliquots to avoid repeated freeze/thaw cycles.

### Other reagents preparation

**Table:** 

Reagent	Final stock concentration	Amount	Volume
DAPI	2 mg/mL	10 mg	5 mL MilliQ

Store at 4°C for up to 3 months.

## Key resources table

**Table:** 

REAGENT or RESOURCE	SOURCE	IDENTIFIER
**Biological samples**		

Human intestinal organoids	Utrecht Medical Center	N/A

C**hemicals, peptides, and recombinant proteins**		

Advanced DMEM-F12	Thermo Fisher Scientific	12634-010
Penicillin-Streptomycin	Thermo Fisher Scientific	15140122
HEPES	Thermo Fisher Scientific	15630080
GlutaMax	Thermo Fisher Scientific	35050061
B-27 Supplement	Thermo Fisher Scientific	17504044
Nicotinamide	Sigma Aldrich	N0636
N-Acetyl-L-cysteine	Sigma Aldrich	A9165
SB202190 (p38 inhibitor)	Sigma Aldrich	S7076
EGF	PeproTech	AF-100-15
A83-01 (ALK4/5/7 inhibitor)	Tocris	2939
Prostaglandin E2	Tocris	2296
Y-27632 dihydrochloride (Rho-kinase inhibitor)	AbMole	M1817
Primocin	Invivogen	ant-pm-2
BMP-2	PeproTech	120-02C
BMP-4	PeproTech	120-05ET
DAPT	Sigma	D5942
IGF-1	PeproTech	100–11
FGF-2	PeproTech	100-18B
Wnt3A surrogate	U-Protein Express	Custom order
Noggin-Fc Fusion Protein conditioned medium	U-Protein Express	Custom order
R-spondin1-conditioned medium	In-house production (see (Pleguezuelos-Manzano et al., 2020)	N/A
Doxycycline monohydrate	Sigma	D1822
DAPI	Thermo Fisher Scientific	D1306
TrypLE™ Express Enzyme (1×), phenol red	Thermo Fisher Scientific	11568856
Bovine serum albumin (BSA)	Sigma	A2153
Dimethyl sulfoxide (DMSO)	Sigma	D8418
Basement membrane extract (BME), Type II	R&D Systems	3533-001-02
Matrigel	Corning	356231

**Critical commercial assays**		

Quick/DNA MicroPrep	Zymo Research	D3021

**Recombinant DNA**		

pSpCas9(BB)-2A-GFP PX458	Ran et al. (2013)	Addgene plasmid 48138

**Software and algorithms**		

Benchling	N/A	https://benchling.com
ICE-Synthego	N/A	https://www.synthego.com/

**Other**		

24-well suspension culture plate	Greiner Bio-One	662102
12-well suspension culture plate	Greiner Bio-One	665102
6-well suspension culture plate	Greiner Bio-One	657185
15 mL conical tube	Greiner Bio-One	188271
50 mL conical tube	Greiner Bio-One	227261
Blue filter lid FACS tube (Falcon™ 352235)	Thermo Fisher Scientific	08-771-23
Electroporation cuvettes	Nepa Gene	EC-OO2S
NEPA21 Super Electroporator	Nepa Gene	N/A
Flow cytometry cell sorter (e.g., BD Influx™)	BD Biosciences	N/A
Fluorescence and brightfield microscope (e.g., EVOS Cell Imaging System)	Thermo Fisher Scientific	N/A

## Materials and equipment

**Table:** ADF+++ (500 mL)

Reagent	Final concentration	Amount
Advanced DMEM-F12	N/A	485 mL
Penicillin-Streptomycin 10,000 U/mL	100 U/mL	5 mL
HEPES 1 M	10 mM	5 mL
GlutaMax 200 mM	2 mM	5 mL

Media can be stored at 4°C for up to 1 month.

**Table:** Expansion medium (EM) (100 mL)

Reagent	Final concentration	Amount
ADF+++	N/A	74.4 mL
Wnt3A surrogate	0.15 nM	100 μL
R-spondin 1-conditioned medium	20 % final volume	20 mL
Noggin-Fc Fusion Protein conditioned medium	2% final volume	2 mL
B-27 Supplement	2% final volume	2 mL
Nicotinamide 1 M	10 mM	1 mL
N-Acetyl-L-cysteine 500 mM	1.25 mM	250 μL
SB202190 (P38 inhibitor) 30 mM	3 μM	10 μL
EGF 500 μg/mL	50 ng/mL	10 μL
A83-01 (TGF-β inhibitor) 5 mM	500 nM	10 μL
Prostaglandin E2 10 mM	1 μM	10 μL
Primocin 50 mg/mL	0.1 mg/mL	200 μL

Media can be stored at 4°C for up to 2 weeks.

**Table:** Modified IF (mIF) medium (100 mL) (Fujii et al., 2018)

Reagent	Final concentration	Amount
ADF+++	N/A	74.3 mL
Wnt3A surrogate	0.15 nM	100 μL
R-spondin 1-conditioned medium	20% final volume	20 mL
Noggin-Fc Fusion Protein conditioned medium	2% final volume	2 mL
B-27 Supplement	2% final volume	2 mL
N-Acetyl-L-cysteine 500 mM	1.25 mM	250 μL
IGF-1 100 μg/mL	100 ng/mL	100 μL
FGF-2 50 μg/mL	50 ng/mL	100 μL
Primocin 50 mg/mL	0.1 mg/mL	200 μL

Media can be stored at 4°C for up to 2 weeks.

**Table:** ENR differentiation medium (100 mL)

Reagent	Final concentration	Amount
ADF+++	N/A	85.5 mL
R-spondin 1-conditioned medium	10% final volume	10 mL
Noggin-Fc Fusion Protein conditioned medium	1% final volume	2 mL
B-27 Supplement	2% final volume	2 mL
N-Acetyl-L-cysteine 500 mM	1.25 mM	250 μL
EGF 500 μg/mL	50 ng/mL	10 μL
Primocin 50 mg/mL	0.1 mg/mL	200 μL
**OPTIONAL**: DAPT 10 mM	10 μM	100 μL

Media can be stored at 4°C for up to 2 weeks.

**Table:** ERBMP differentiation medium (100 mL)

Reagent	Final concentration	Amount
ADF+++	N/A	87.3 mL
R-spondin 1-conditioned medium	10% final volume	10 mL
BMP-2 50 μg/mL	50 ng/mL	100 μL
BMP-4 50 μg/mL	50 ng/mL	100 μL
B-27 Supplement	2% final volume	2 mL
N-Acetylcysteine 500 mM	1.25 mM	250 μL
EGF 500 μg/mL	50 ng/mL	10 μL
Primocin 50 mg/mL	0.1 mg/mL	200 μL
**OPTIONAL**: DAPT 10 mM	10 μM	100 μL

Media can be stored at 4°C for up to 2 weeks.

**Table:** Fluorescence-activated cell sorting (FACS) buffer (10 mL)

Reagent	Final concentration	Amount
ADF+++	N/A	10 mL
Y-27632 dihydrochloride 10 mM	10 μM	10 μL
DAPI 2 mg/mL	0.2 μg/mL	10 μL

Prepare within 24 h before use and store at 4°C.

## Step-by-step method details

### Maintenance of hIOs

**Timing: 1 h per week**

In this section, we explain how hIO cultures can be expanded. This method applies to hIOs established from any part of the small intestine. The establishment of hIOs from intestinal tissue is described elsewhere (Sato et al., 2011; Fujii et al., 2015; Pleguezuelos-Manzano et al., 2020).

**CRITICAL:** It is important to keep the basement extracellular matrix (BME) or Matrigel on ice at all times during culture. Store it at −20°C and thaw it on ice or at 4°C before use. Similarly, tubes containing hIOs should be kept on ice in-between steps.

**Figure 1:**
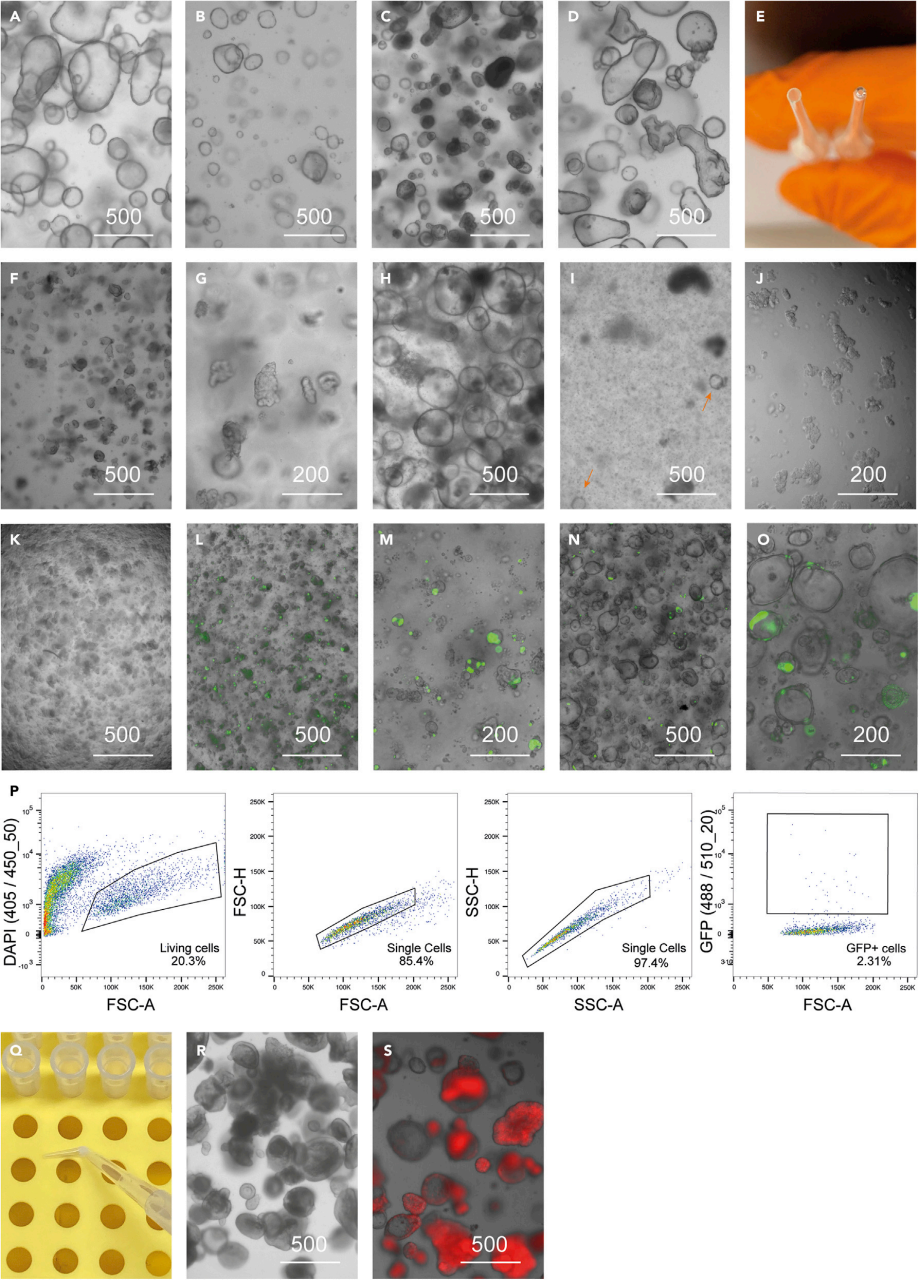
Brightfield and fluorescence-microscopy images of hIOs in different growth states and upon electroporation (A–C) (A) Brightfield images of hIOs 5 days after passage and plated at an optimal density, (B) plated too sparsely, (C) plated too densely and thus presenting a necrotic core. (D) hIOs starting to spontaneously differentiate. (E) Glass Pasteur pipette before (left) and after (right) being narrowed to an appropriate diameter. (F and G) Brightfield image of dissociated hIOs with optimal size for passaging. (H and I) (H) Transduced hIOs before selection and (I) after selection. (J) Brightfield image of dissociated hIOs of approximately 10 cell clumps, optimal for electroporation. (K) Plated hIOs immediately after electroporation. (L–O) (L and M) GFP+ hIOs one day after electroporation and (N and O) 5 days after electroporation. (P) Gating strategy for sorting GFP+ living (DAPI negative) single cells using flow cytometry. (Q) Bent pipette tip for organoid picking. (R) Brightfield image of hIOs on day 5 of ENR differentiation. (S) EEC-enriched hIOs (dTomato positive) after ENR + doxycycline differentiation (day 5). Scale bar in μm.

***Note:*** hIOs are grown in a humidified incubator at 37°C with 5% CO_2_. Culture plates should be prewarmed at 37°C (e.g. in the incubator) one day before plating hIOs. Most hIOs can be passaged every 5–7 days with 1 to 4 or 5 split ratio. See Figure 1A for hIOs ready to be passaged.

1. Remove media from desired wells, flush BME or Matrigel (gel) droplets containing hIOs using 1 mL ice-cold ADF+++.
2. Aspirate and dispense cold ADF+++ repeatedly until gel droplets are dissolved and collect in a 15 mL conical tube.
3. Pipette the mixture up and down ∼10 times inside the tube to further disrupt gel.
4. Centrifuge hIOs at 500 x g for 5 min at 4°C.
5. In the meantime, produce a narrowed glass Pasteur pipette using a Bunsen burner. This can be achieved by holding the glass pipette tip into the fire for a few seconds while quickly rotating the glass pipette.

***Note:*** This should result in a reduction of the diameter of the hole to roughly about one third of its original size, approximately 0.3 mm wide (see Figure 1E for an example of a narrowed glass pipette).

6. After centrifugation, the gel will appear as a cloudy layer on top of the hIOs. Aspirate supernatant and remove as much gel as possible.

***Optional:*** If a clear separation between hIOs and gel cannot be observed, it is likely the gel is not sufficiently disrupted. In this case, remove the supernatant, resuspend the pellet in 10 mL ice-cold ADF+++ and repeat centrifugation.

7. Add 1.5 mL ice-cold ADF+++ to the tube containing the organoid pellet.
8. Sterilize the narrowed glass pipette with the Bunsen burner, attach it to a Pipette controller, aspirate and dispense 2 mL of ice cold ADF+++ twice.

***Note:*** This cools down the glass pipette and precoats its surface to avoid sticking of hIOs and material loss.

9. Next, use this glass pipette to pipette the organoid solution up and down 10–20 times. The glass pipette can be placed against the bottom of the tube when pipetting the organoid solution to create some resistance.

***Note:*** Organoid dissociation ease will depend on the organoid line, the size of the glass pipette and the velocity of pipetting. More cystic hIOs will generally be easier to dissociate.

10. Pipette ∼10 μL of hIOs into a plate to observe size under the microscope. hIOs should be dissociated into approximately 20-cells clumps (see Figures 1F and 1G).

***Note:*** Too small fragments, including single cells, will result in reduced outgrowth efficiency. Larger fragments will not allow optimal splitting and expansion ratios, and might necessitate premature passaging.

11. Centrifuge organoid fragments at 500 x g for 5 min at 4°C.
12. Aspirate the supernatant, add gel and resuspend.
13. Select a well plate of choice and plate the recommended volume of gel per well (see Table 1).

**Table 1:** Recommended gel volumes and media volume per type of well plate

	24-Well plate	12-Well plate	6 well plate
Gel volume / well	50 μL	100 μL	200 μL
Media volume / well	0.5 mL	1 mL	2.5 mL

***Optional:*** Gel can be mixed 3:1 with ice cold ADF+++ to reduce the use of gel.

14. Plate hIOs in droplets of approximately 10–20 μL.
15. Turn the plate carefully upside down and transfer to the incubator (this will prevent hIOs from sinking to the bottom of the gel, which might result in partial 2D growth).
16. Let the droplets solidify for 15–30 min.
17. Carefully add prewarmed media to wells.
18. Refresh the media every 2–3 days.

### Lentiviral transduction to generate doxycycline-inducible NEUROG3 overexpressing hIOs

**Timing: 2 weeks**

This section is facultative to the protocol, and describes the lentiviral transduction of hIOs. Lentiviruses carrying the inducible overexpression of NEUROG3 can be employed for the production of high-purity EEC cultures. The targeting construct encoding doxycycline-inducible NEUROG3 expression is described elsewhere (Addgene 75337, Zhu et al., 2016; or a pLX-based vector in Beumer et al., 2020). The pLX-based system encodes a constitutively expressed transactivator (rtTA) incorporated in the plasmid. The strategy by Zhu et al. makes use of a separately-delivered rtTA-expressing transgene. Production of lentiviral particles is described elsewhere (Tiscornia et al., 2006).

**CRITICAL:** When working with lentiviral particles and lentivirus-infected hIOs, it is necessary to follow biosafety level 2 guidelines.

***Note:*** Lentiviral batches produced on a 15-cm dish containing 293T cells (50–80% confluency) generally generate sufficient viral particles for at least five transductions.

19. After concentration of lentiviral supernatant by ultracentrifugation (Tiscornia et al., 2006) dissolve the viral pellet in 500 μL infection medium (EM supplemented with 10 μM Y-27632 and 10 μg/mL Polybrene).

**Pause point:** Lentiviruses can be used immediately or stored long-term at −80°C in 500 μL infection medium in a cryovial.

20. About 5–7 days before transduction, passage hIOs as usual, 1 or 2 wells of a 12-well plate (equivalent to 100–200 μL of gel) are necessary for each transduction reaction.
21. Immediately before transduction, prewarm TrypLE solution supplemented with 10 μM Y-27632 at 37°C.
22. Remove media from desired wells.
23. Flush hIOs from the bottom of the well using 1 mL ice-cold ADF+++ and collect in a 15 mL conical tube.
24. Pipette the mixture up and down ∼10 times inside the tube to disrupt gel.
25. Centrifuge hIOs at 500 x g for 5 min at 4°C.
26. After centrifugation, the gel will appear as a cloudy layer on top of the hIOs. Aspirate supernatant and remove as much gel as possible using a pipette.

***Optional:*** If a clear separation between hIOs and gel cannot be observed, remove the supernatant, resuspend the pellet in 10 mL ice-cold ADF+++ and repeat centrifugation.

**CRITICAL:** It is crucial to remove as much gel as possible as it might hamper the following dissociation process.

***Note:*** hIOs are very sticky at this point and will easily attach to tube walls and pipette tips. To avoid excessive loss of material, coat pipette tips with TrypLE during the following step (and with ADF+++ after dissociation) by pipetting the solutions up and down a couple of times.

27. Precoat a P1000 filter pipette tip in TrypLE before resuspending hIOs in 2 mL of prewarmed TrypLE solution.
28. Incubate hIOs in TrypLE for 3–5 min in a 37°C water bath.

**CRITICAL:** TrypLE causes cell death and organoid clumping at the bottom of the tube. Flick the tube containing the hIOs while they are incubating in the water bath to prevent them from sedimenting and clumping.

29. Using a narrowed Pasteur glass pipette (see step 5), precoated in TrypLE, forcefully pipette up and down to disrupt hIOs. For transduction, hIOs are dissociated to single cells.
30. Monitor the dissociation level under the microscope.

***Note:*** If single cells are not obtained after 5 min of TrypLE incubation and subsequent mechanical dissociation, clumps can be subjected to a maximum of 5 min additional incubation.

31. To inactivate TrypLE, resuspend the mixture in 10 mL cold ADF+++ supplemented with 10 μM Y-27632 and centrifuge at 300 x g for 5 min at 4°C.
32. Aspirate the supernatant and resuspend the cell pellet in 150 μL infection medium (EM supplemented with 10 μM Y-27632 and 10 μg/mL Polybrene).

***Optional:*** If viral titer and subsequent copy number integration are vital for further experimentation, different concentrations of lentiviruses can be used for transduction.

33. Combine 150 μL of the resuspended cells with 100 μL of concentrated lentiviruses in one well of a 48-well plate and mix well by pipetting up and down.
34. Wrap the plate in parafilm and spin cells down at 600 x g for 60 min at 32°C.
35. Remove parafilm and further incubate the cells for 5–8 h at a 37°C incubator.
36. Collect the cells in a 1.5 mL microcentrifuge tube and spin down 300 x g for 5 min at 4°C.
37. Discard the infection medium and resuspend pellet in ∼100 μL gel, mixing well and carefully to avoid bubbles.
38. Plate hIOs in a prewarmed 12 well plate and transfer to the incubator.
39. Once the gel has solidified (about 15 min later) carefully add prewarmed EM supplemented with 10 μM Y-27632.
40. After 3 days of transduction, cells can be selected (Figure 1H shows hIOs prior to selection).
41. The targeting vector encoding inducible NEUROG3 contains a Puromycin resistance cassette. Treat cells for at least 1 week with 1 μg/mL Puromycin.

***Note:*** Cells that survive after one week of Puromycin treatment are effectively transduced with the targeting vector and can be used for further experimentation. An example of hIOs growing out on the background of negatively selected cells can be seen in Figure 1I.

42. Passage the selected hIOs.

***Note:*** Once expanded the hIOs are ready for downstream analysis (see “differentiation” section) and long-term storage by cryopreservation (see Pleguezuelos-Manzano et al., 2020 for information about cryopreservation of hIOs).

***Optional:*** If desired, clonal lines can be established with different lentiviral copy integration. This can be achieved by FAC-sorting selected cells (see below). When transduction efficiency is exceedingly low, individual hIOs that grow out can also be assumed to be derived from a single transduced cell and picked for further clonal expansion (see clonal line generation protocol). Transduction of lentiviruses is generally efficient in hIOs with little protocol-induced cell death. Depending on the viral titer (we do not calculate titer but use different dilutions of viral supernatant) of the NEUROG3-coding targeting vectors, we observe 1% to over 50% transduction efficiency. When puromycin treatment is initiated, cell death should occur as soon as 2–3 days. After 1-week on puromycin, non-transduced cells should be negatively selected.

### Electroporation

**Timing: 1.5–2 h**

In this section we describe how to electroporate hIOs. This protocol works in combination with a fluorescent-based selection method (described below). Other methods for selection of successfully electroporated hIO cells (such as antibiotic selection or growth factor withdrawal) will require modifications of this strategy or the use of alternative protocols.

**CRITICAL:** Multiple steps of the electroporation and clonal line generation protocols are performed under non-sterile conditions; it is thus necessary to use both Penicillin-Streptomycin as well as primocin in the media to prevent the contamination of the cultures.

43. About 5–7 days before the electroporation, passage hIOs as usual.

***Note:*** 200–400 μL gel containing hIOs (e.g. 2–4 wells of a 12-well plate) is required for each electroporation reaction.

**CRITICAL:** It is essential for the efficiency of electroporation that the hIOs are growing cystically and have not started differentiation (see Figure 1A for cystic growing hIOs and 1D for hIOs that have started differentiation).

44. 24h before electroporation, add fresh media supplemented with 10 μM Y-27632 and 1.25% vol/vol DMSO, thaw gel at 4°C.
45. Immediately before electroporation, prewarm TrypLE solution supplemented with 10 μM Y-27632 at 37°C.
46. Dissociate hIOs as described before (step 27–29), to obtain clumps of 2 to 20 cells (see Figure 1J). Closely monitor the morphology of the hIOs under the microscope until the desired fragment size is achieved.
47. To inactivate TrypLE, resuspend the mixture in 10 mL cold ADF+++ supplemented with 10 μM Y-27632 and centrifuge at 300 x g for 5 min at 4°C.
48. Aspirate supernatant and resuspend organoid pellet in 6 mL cold Optimem, centrifuge as in previous step and repeat Optimem wash.
49. In the meantime, prepare 1.5 mL microcentrifuge tubes containing 15 μg of the Cas9 gRNA vector (1 per reaction).
50. Aspirate the supernatant.
51. Resuspend hIOs in Optimem, use 100 μL per cuvette or reaction. Transfer 100 μL of cells with Optimem to the microcentrifuge tube containing DNA, mix.
52. Transfer the cell and DNA mixture to the cuvette and immediately proceed to the next step to avoid cell clumping.
53. Adjust electroporator settings (Table 2).

**Table 2:** Electroporation settings

	Poring pulse	Transfer pulse
Voltage	175 V	20 V
Pulse length	5 ms	50 ms
Pulse interval	50 ms	50 ms
Number of pulses	2	5
Decay rate	10%	40%
Polarity	+	+/−
54. Use the electroporator to measure impedance.

***Note:*** This value should be between 25 and 55 Ω for best results (refer to the troubleshooting guide for more information).

55. Electroporate cells.
56. Add 1 mL ADF+++ supplemented with 10 μM Y-27632 to the cuvette. Precoat the provided Pasteur pipette bulb with ADF+++ and use it to transfer cells to a 1.5 mL microcentrifuge tube.
57. Let the cells recover at room temperature for 30 min.
58. Centrifuge cells at 500 x g for 5 min at 4°C.
59. Discard the supernatant and resuspend the pellet in gel mixing well and carefully to avoid bubbles. Plate hIOs in a prewarmed 12 well plate.

**CRITICAL:** Plating cells at high density greatly increases the cell viability and the overall efficiency. Aim for 100 μL of gel for every 300 μL of starting material. See Figure 1K for hIOs plated immediately after the electroporation.

60. Once the gel has solidified, about 15 min later, carefully add prewarmed EM supplemented with 10 μM Y-27632 and 1.25% vol/vol DMSO.
61. The day after the electroporation, check cells under a fluorescent microscope to assess efficiency.

***Note:*** About 5% of the cells should be GFP-positive at this point (see Figures 1L and 1M).

62. Refresh wells with prewarmed EM supplemented with 10 μM Y-27632.

### Fluorescence-activated cell sorting (FACS)

**Timing: 1.5–2 h**

Three to four days after the electroporation GFP positive cells can be sorted by FACS. Figures 1N and 1O show hIOs before the dissociation.

63. Harvest electroporated hIOs and non-electroporated hIOs to use as negative control.
64. Incubate in TrypLE as described in steps 27–29. Closely monitor the dissociation process under the microscope.

***Note:*** Dissociation is successful when most of the hIOs have broken down into single cells. Complete dissociation of all cellular fragments into single cells is not expected. Longer incubation will yield more single cells but drastically reduce cell viability.

65. To inactivate TrypLE, resuspend the mixture in 10 mL cold ADF+++ supplemented with 10 μM Y-27632 and centrifuge at 500 x g for 5 min at 4°C.
66. In the meantime, prewet the filtered cap of a 5 mL FACs tube with 100 μL of ADF+++ supplemented with 10 μM Y-27632.
67. Aspirate the supernatant carefully and resuspend the cell pellet in 400 μL cold FACS buffer (see “media preparation” section).
68. Using a P1000 filter pipette tip transfer cells through the filter FACS tube. If necessary, pipette up and down while pressing the filter to achieve this.
69. Prepare the necessary 1.5 mL microcentrifuge tubes containing 100 μL ADF+++ supplemented 10 μM Y-27632 to collect the sorted cells.
70. Sort single living cells based on GFP expression (see Figure 1P). Use negative control to apply correct gating strategies.

***Note:*** Outgrowth efficiency ranges from ∼1% to above 20% in different organoid lines. A minimum of 300-500 cells are necessary to achieve a sufficient number of clonal hIOs, 1000–3000 cells are most optimal.

71. Centrifuge cells and remove approximately 80 μL of supernatant carefully using a p200 pipette.

***Optional:*** In case very large numbers of cells are sorted, remove any additional excess volume added through sorting to leave approximately 20 μL of cells-containing liquid.

**CRITICAL:** It is essential to remove the supernatant carefully and slowly to avoid losing the cell pellet, which will not be visible.

72. Resuspend hIOs in gel. Use 50 μL of gel per 500 cells.
73. Dispense hIOs into a prewarmed 24-well or 12-well plate.
74. Allow gel to solidify for 15 min.
75. Add prewarmed EM supplemented with 10 μM Y-27632.
76. Refresh wells with EM supplemented with 10 μM Y-27632l every 2–3 days until hIOs are ready for clonal line generation.

**CRITICAL:** it is important to add media frequently (every 3 days) as evaporation is detrimental for outgrowth.

### Clonal line generation and genotyping

**Timing: 3 days**

In this section we explain how to generate clonal lines from the electroporated bulk and sorted population and how to determine whether each one has been successfully gene-edited.

***Note:*** Once hIOs have reached a diameter of around 250 μm, usually about 10–14 days after sorting, these can be individually passaged and transferred to a new well to generate clonal organoid lines that will be subsequently genotyped.

77. Prewarm TrypLE solution supplemented with 10 μM Y-27632 at 37°C.
78. Bend a p10 pipette tip to a 30° angle (see Figure 1Q).
79. Working under the microscope, use the bent pipette tip to pick an individual organoid from the gel and transfer it to a 1.5 mL microcentrifuge tube.

***Optional:*** If the hIOs are too densely plated, picking a single organoid can be challenging. In that case, transfer a small clump of hIOs to a 10 cm plate and from there pick an individual organoid.

**CRITICAL:** It is important to pick hIOs that have not started spontaneous differentiation or that have visibly fused to other hIOs, interfering with their clonality. Spontaneous differentiation can be identified by changes in hIO morphology, such as thickening of the cell layer, reduction of lumen size and budding (see Figure 1D).

80. Repeat the previous step for 10 to 20 clones.

***Note:*** The optimal number of picked organoids will depend on the targeting efficiency of each gRNA and the outgrowth efficiency after picking, we recommend to pick at least 20 hIOs per gRNA.

81. Add 100 μL of TrypLE solution with 10 μM Y-27632 to each microcentrifuge tube containing the individual hIO.
82. Place microcentriuge tubes containing the hIOs in an appropriate rack and transfer rack to a 37°C incubator. Incubate hIOs in TrypLE for 30 min.
83. To dissociate hIOs, vortex each tube 10–20 s at 3000 rpm, monitoring dissociation process under the microscope.

***Note:*** Clumps of ∼10 cells give best results.

84. Add 1 mL ADF+++ supplemented with 10 μM Y-27632 to each microcentrifuge tube to inactivate TrypLE.
85. Centrifuge cells at 600 x g for 10 min at 4°C.
86. Aspirate supernatant very carefully not to disrupt pellets of cells. Leave about 20 μL of media in the microcentrifuge tube.

**CRITICAL:** It is important to remove the supernatant carefully; the pellet of cells cannot be observed by the naked eye but should still be visible by placing the microcentrifuge tube directly under the microscope.

87. Add 30 μL gel and plate the material in a numbered well of a 24-well plate. Keep ∼5 μL in the (also numbered) microcentrifuge tube.
88. After plating all clones, keep the remaining material inside the microcentrifuge tube, this can be used for genotyping.

**Pause point:** The cell pellet can be used for genotyping immediately or maintained at −20°C until necessary.

89. Isolate genomic DNA (gDNA) using a commercial gDNA isolation kit according to the manufacturer’s instructions, always making sure to centrifuge the empty column before eluting the gDNA in order to remove as much wash buffer as possible. Elute in 12 μL MilliQ water.

***Note:*** Some gDNA isolation kits (such as the zymogen gDNA isolation kit) can be used to isolate sufficient gDNA from little starting material (10–100 cells). Depending on the kit requirements, the starting material may need to be increased by first plating all the cells in step 88 and after these have grown into hIOs collecting some material at multiple locations of the well. Depending on the kit, the elution volume may need to be adjusted.

**Pause point:** The isolated gDNA can be used immediately or stored short-term at 4°C or long-term at −20°C.

90. Prepare a PCR master mix with the following components for the genomic DNA amplification.

**Table:** PCR reaction master mix

Reagent	Volume
DNA template	5 μL
DNA Polymerase	0.25 μL
Primer 1 10 uM	0.5 μL (final 0.1 μM)
Primer 2 10 uM	0.5 μL (final 0.1 μM)
dNTPs	1 μL
MgCl2	5 μL
GoTaq green master mix 5×	10 μL
ddH_2_O	27.75 μL

91. Mix PCR master mix and DNA in a PCR tube.
92. Centrifuge PCR strip or tubes.
93. Perform PCR amplification.

**Table:** PCR cycling conditions

Steps	Temperature	Time	Cycles
Initial denaturation	95°C	2 min	1
Denaturation	95°C	30 s	35 cycles
Annealing	55°C–61°C	30 s	
Extension	72°C	30 s per 500 bases	
Final extension	72°C	10 min	1
Hold	4°C	Forever

***Note:*** Cycling temperature and times may need to be adapted based on the reagents used. The annealing temperature will depend on the primers and should be adjusted accordingly. Similarly, the extension step length depends on the size of the sequence to be amplified.

94. Load 10 μL of the PCR reaction in a 2% agarose gel electrophoresis and run for 30–50 min at 90V in TAE buffer.
95. Image gel.
96. If a clear single band of the correct size is observed, proceed with PCR clean-up according to manufacturer’s instructions, elute samples in 15 μL MilliQ.

***Note:*** It is possible that in some samples, the two alleles have different sizes due to large insertions and deletions, which manifests as multiple bands. If, however, multiple bands are observed in all reactions, it is probable that this occurs due to unspecific amplification.

***Optional:*** If more than two bands are observed in the agarose gel, test another primer pair. If also unsuccessful, try adjusting PCR parameters or load the remaining PCR reaction in an agarose gel, cut the band of the appropriate size and perform agarose gel purification (see troubleshooting guide for more information about genotyping).

**Pause point:** PCR clean-up products can be stored short-term at 4°C or long-term at −20°C

97. To precisely detect introduced insertions and deletions (indels), it is necessary to evaluate the PCR amplicons by Sanger sequencing.
98. Use Benchling or other visualization software to assess the Sanger sequencing results.

**Figure 2:**
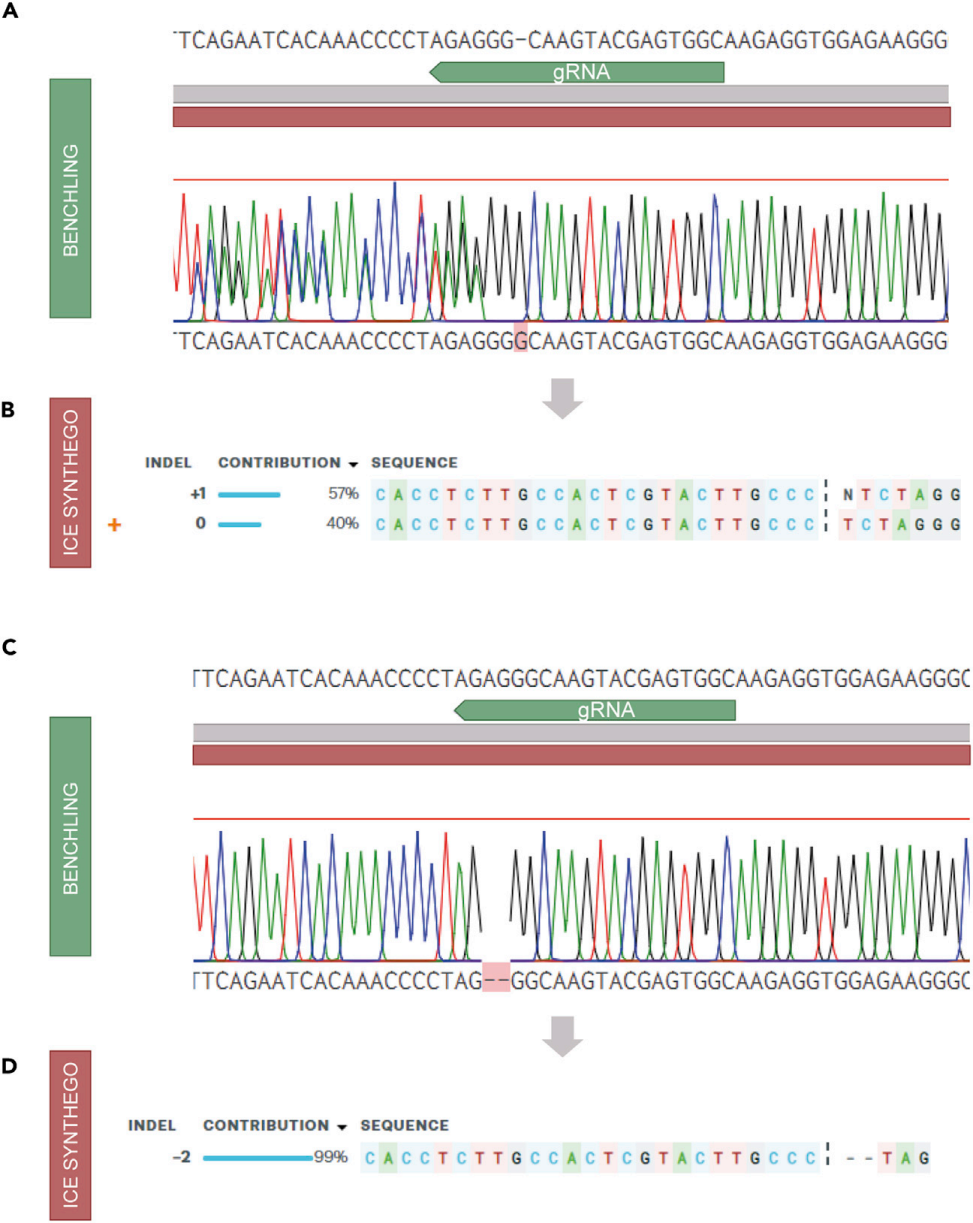
Examples of organoid gDNA sequencing results (A and C) Sanger sequencing results of a heterozygous (A) and homozygous (C) mutant clone as seen in Benchling. (B and D) ICE Synthego results of the deconvolution of the Sanger sequences.

***Note:*** In most cases, to be able to determine whether the mutant clones contain out-of-frame homozygous mutations, it is necessary to deconvolute the two allelic sequences. ICE Synthego and TIDE (Brinkman et al., 2014) are free online CRISPR efficiency assessment tools that perform such analysis. Figure 2A shows a mixed allele sequence in Benchling. When deconvoluted using ICE Synthego (Figure 2B), this reveals two alleles, one presenting 1 bp insertion and the other being wild-type. It is also possible both alleles are mutant but contain different indels also appearing as a mixed allele sequence. Figures 2C and 2D show the sequencing results of a sample containing the same mutation in both alleles.

**CRITICAL:** Indels that are divisible by three (such a 6-bps deletion or a 9-bps insertion) will not result in a frameshift.

99. Passage the selected knockout clones and a couple of wild-type clones to use as control.

***Note:*** Once sufficiently expanded, the hIOs are ready for downstream analysis and long-term storage by cryopreservation (see Pleguezuelos-Manzano et al., 2020 for information about cryopreservation of hIOs).

### Differentiation

**Timing: 8–10 days**

In this section, we outline how hIOs can be differentiated towards effector cell types. Modified IF (mIF) effectively introduces a mixture of ASC, progenitors and differentiated cells including absorptive enterocytes, the secretory lineage (goblet cells, EECs) and microfold cells (Fujii et al., 2018). This condition best resembles the diversity of cell types in the human intestine. However, many research questions demand only (specific) effector cell types. For general differentiation towards (crypt) and bottom villus enterocytes, goblet cells and EECs, growth factor-reduced ENR can be used (Sato et al., 2011). To trigger functional specialization and induce the villus (tip) phenotype of all these cell types, we recommend stimulation of BMP signaling with ERBMP (Beumer et al., 2018, 2022; Moor et al., 2018). For both ENR and ERBMP, the addition of the NOTCH-inhibitor DAPT (further) enhances differentiation towards the secretory lineage (Beumer and Clevers, 2021). Finally, rare EECs are efficiently introduced by overexpression of NEUROG3, which results in an enrichment of 40–80% of EECs (Beumer et al., 2020). The advised duration of differentiation is 5 days for all these protocols. This roughly reflects the lifetime of intestinal cells that transit from the crypt bottom to the villus tip and that differentiate along this journey. Longer exposure (7 days) increases the efficiency, but at the cost of increased cell death.

100. Culture hIOs in EM for 3–5 days post-split as described above (section “maintenance of hIOs”) to grow these to sufficient size (see Figure 1A).

***Optional:*** Use hIOs with doxycycline-inducible NEUROG3 overexpression (previously established using section “lentiviral transduction to generate doxycycline-inducible NEUROG3 overexpressing hIOs” combined with steps 102.f or 102.g to highly efficiently produce EECs.

101. Remove EM and incubate hIOs in pre-warmed ADF+++ for 30 min at 37°C to wash out remaining growth factors.
102. Remove ADF+++ and add pre-warmed differentiation medium of choice:
  a. mIF.
  b. ENR.
  c. ENR + 10 μM DAPT.
  d. ERBMP.
  e. ERBMP + 10 μM DAPT.
  f. If inducible NEUROG3 overexpressing hIOs are used:ENR + 250 ng/mL doxycycline for 2 days, followed by ENR for 3 days.
  g. If inducible NEUROG3 overexpressing hIOs are used:ERBMP + 250 ng/mL doxycycline for 2 days, followed by ERBMP for 3 days.
103. Incubate hIOs in differentiation medium at 37°C for 5 days.
104. Refresh the medium every 2 days. See Figure 1R for hIOs grown in ENR, and Figure 1S for hIOs grown in ENR + doxycycline.
105. Collect hIOs for downstream assays of choice (e.g., quantitative PCR, RNA sequencing, immunocytochemistry/-histochemistry).

## Expected outcomes

Most hIOs can be passaged every 5–7 days with 1 to 4 or 5 split ratios.

In the course of the electroporation protocol, considerable cell death is expected (see Figures 1L and 1M). Green fluorescent signal arises 14–18 h after electroporation (see Figures 1L and 1M). GFP-positive cells can be sorted 3–4 days after the electroporation. Depending on the efficiency of the process the percentage of sorted GFP positive cells can range between 1-20%. The total numbers should exceed 500 in order to obtain sufficient outgrowth, this number can be also influenced by the dissociation efficiency. After 10 days approximately, clonal lines can be generated by picking individual hIOs and transferring them to a new well. We recommend to start by picking 24 clones, as some of these (5–10%) can be lost during the process. Organoid genotyping sometimes requires testing multiple sets of PCR amplification primers and sequencing primers. To save time, primers can also be tested in advance. Targeting efficiency varies widely for different genes and gRNAs, but in our experience most electroporation attempts yield at least 2 homozygous mutant clones out of the 24 picked clones, with only a few cases in which no clones contained the mutation of interest.

During the first 2 days of differentiation, the hIOs will undergo morphological changes and an increase in cell death. In the case of doxycycline-induced hIOs, fluorescent signal can be observed after 12 h. The NEUROG3 induction efficiency - determined by dTomato levels - lies between 50-80% if overexpressing in a homogenous stem/progenitor cell population. If organoids are more differentiated when overexpression is initiated, efficiency can drop to almost 0%. Changes in cell type composition, the transcriptome and protein expression can be found in the original papers describing the various differentiation conditions (Sato et al., 2011; Fujii et al., 2018; Beumer et al., 2020, 2022; Beumer and Clevers, 2021).

## Limitations

Although hIOs capture many features of native epithelium, some aspects are currently not modeled. Multiple morphogen gradients exist from crypt to villus, patterning cell states along this axis. For example, BMP signaling dictates hormone expression in the crypt versus villus compartment. Organoid cultures currently do not allow for capturing all these different cell states at the same moment. Signaling pathway activity can be controlled through the addition of different concentrations of certain agonists or antagonists, allowing the induction of individual cell states and their transition zones. Technological advances such as engineered gut tubes with different medium flow channels would allow mirroring morphogen gradients. Compared to mouse small intestinal organoids, there are currently no protocols that produce ratios of dividing progenitor and mature cell types matching those of tissue.

Enteroendocrine numbers are extremely low in tissue (below 1% of the total cell number), and therefore remain a particularly difficult cell type to study. We provide multiple strategies to increase their numbers: (1) the enrichment of secretory lineage by the addition of NOTCH inhibitor; (2) an inducible NEUROG3 overexpression system via lentiviral transduction of the hIOs. The latter results in a large increase of the total number of EECs (up to 80%). However, the increase in EECs comes at the expense of other cell types, which diverts the composition from *in vivo* ratios. As a result, the optimal differentiation media should be selected according to the question of interest. It is also important to note that the efficiency of the differentiation protocol can vary between experiments, depending on the growth state of hIOs.

We provide a protocol for the generation of clonal knockout lines by CRISPR-Cas9 through the introduction of DSBs. A major drawback of this protocol is the duration, up to 3 months, and extensive hands-on labor. Unspecific gene editing can always occur. To minimize the effect, we recommend comparing multiple knockout clones. Finally, for modeling of specific mutations, CRISPR-Cas9 base editors or prime editors are better suited. These strategies can be more specific and cause less DNA damage but require more extensive cloning and tend to be less efficient.

Finally, ASC-derived organoids in the described setup only allow modeling of epithelial processes. For example, lack of an enteric nervous system precludes the study of interactions between epithelial EECs and neurons. Transfer of packaged lipids from enterocytes to surrounding lymph vessels cannot be assessed. To start addressing some of these biological processes that stretch beyond the epithelium, this reductionist system can be supplemented with co-cultures of relevant cell types.

## Troubleshooting

### Problem 1

Poor organoid growth during organoid maintenance.

### Potential solution

If the hIOs are seeded too sparsely (Figure 1B), growth can potentially be impaired. This is often accompanied by cell death and differentiation. If hIOs are seeded too densely (Figure 1C), a necrotic core of dead hIOs will be visible in the middle of the dome due to lack of growth factors. See Figure 1A for proper seeding density. It is also important to use organoid media not older than one month.

### Problem 2

Ineffective differentiation.

### Potential solution

It is crucial that the hIOs are growing cystically before differentiation, representing a population of uncommitted, multipotent progenitor cells.

### Problem 3

Ineffective differentiation: no BMP effect is observed.

### Potential solution

Completely remove Noggin before adding the BMP ligands. To do this, first aspirate EM and then incubate hIOs in pre-warmed ADF+++ for 30 min at 37°C to wash out remaining growth factors. Then, add differentiation media with BMPs. Avoid free-thaw cycles when using BMP ligands.

### Problem 4

Ineffective transduction.

### Potential solution

Ensure the 293T cells are seeded in a proper density and that the plasmids used for the transfection have a concentration of at least 500 ng/μL.

### Problem 5

Too much cell death is observed during the NEUROG3 overexpression (see Figure 1S for comparison).

### Potential solution

Lower the doxycycline concentration. High copy number integration could be associated with a more efficient overexpression of the transcription factor.

### Problem 6

During the electroporation, the impedance measured by the electroporator is too low (below 25 Ω) or too high (more than 55 Ω).

### Potential solution

Reduce impedance by increasing Optimem volume. To increase impedance, it is necessary to increase the organoid starting material.

### Problem 7

After the electroporation, too much cell death can be observed (see Figure 1L for comparison).

### Potential solution

Make sure to use good quality plasmid for the electroporation and prevent salt contamination. During the plasmid preparation and alcohol precipitation, elute the plasmids in MilliQ water after all ethanol has evaporated. The plasmids should have a concentration of approximately 1000 ng per μL or more. Increase the organoid starting material and make sure not to TrypLE the cells for too long. After the electroporation, plate cells at very high density (see Figure 1K).

### Problem 8

Too low number of cells during the FACs sorting after electroporation (less than 500).

### Potential solution

Outgrowth efficiency varies per line and gRNA targeting efficiency. Aim to plate 1000–2000 cells per 100 μL, and a total of at least 300 cells. If insufficient numbers, increase starting material. Ensure the hIOs have been dissociated into mostly single cells before sorting.

### Problem 9

There is more than one band visible in all the reactions of the agarose gel during the genotyping step.

### Potential solution

If there are only a few off-target products, in our experience, performing PCR purification and sending purified DNA for sequencing using a separate sequencing primer tends to give good genotyping results. If this fails and there are only a few unspecific bands well distanced from each other, load remaining PCR reaction in an agarose gel, cut band of the appropriate size and perform agarose gel purification. If this also fails or there are too many bands in the agarose gel to precisely cut the band of interest, perform PCR reaction using a different primer pair. It sometimes helps to increase the PCR annealing temperature to 62°C. If also unsuccessful, perform a nested PCR reaction.

### Problem 10

Sanger sequencing results are noisy.

### Potential solution

Make sure there is sufficient DNA after PCR purification (at least 15 ng/μL) and that DNA has been eluted in MilliQ during the PCR purification protocol. Make sure to use a sequencing primer inside the PCR amplification region. It is possible to also test a different sequencing primer. Organoid clones can also be expanded one or more passages to generate a larger amount of genomic DNA, allowing better genotyping.

### Problem 11

Inefficient gene modification: no homozygous mutants can be generated.

### Potential solution

A different gRNA could be employed targeting the same gene, preferentially targeting alternative exons. Due to unpredictable reasons (for example, off-target effects, closed chromatin), on-target efficacy of some gRNAs are low. In case genes are targeted essential for cell survival, gene knockout will be low and conditional genetic modifications are desired.

## Resource availability

### Lead contact

Further information and requests for resources and reagents should be directed to and will be fulfilled by the lead contact, Hans Clevers (h.clevers@hubrecht.eu).

### Materials availability

This study did not generate new unique reagents.

## Data Availability

This study did not generate/analyze dataset/code.
